# Supplementary material for: ANANSE: an enhancer network-based computational approach for predicting key transcription factors in cell fate determination
Source: Nucleic Acids Res. 2021 Jul 9;49(14):7966–85. doi: 10.1093/nar/gkab598 (PMC8373078; doi:10.1093/nar/gkab598)
Supplement: gkab598_Supplemental_Files [file gkab598_supplemental_files.zip › 20210531.Xu_et_al.supplementary_material.revision.v1.3.pdf]

Supplementary Material

ANANSE: An enhancer network-based computational approach for predicting key transcription factors in cell fate determination

Quan Xu, Georgios Georgiou, Siebren Frölich, Maarten van der Sande, Gert Jan C. Veenstra, Huiqing Zhou and Simon J. van Heeringen

Additional file 1: Figure S1. Comparison of ANANSE TF binding prediction performance to other methods. **Figure S2.** Evaluation of tissue-specific enhancer GRNs predicted by ANANSE. **Figure S3.** Evaluation of predicting tissue-specific GRNs based on enhancer, promoter or expression data. **Figure S4.** Evaluation of tissue-specific enhancer GRNs with enhancer-gene summarization methods. **Figure S5.** Comparison of the top 10 key TFs predicted by different GRN sizes in seven experimentally validated trans-differentiation strategies. **Figure S6.** Comparison of different GRN sizes used in the ANANSE prediction in seven experimentally validated trans-differentiation strategies. **Figure S7.** Comparison of the top 10 key TFs predicted by different GRNs (enhancer, promoter and expression) in seven experimentally validated trans-differentiation strategies. **Figure S8.** Evaluation of the performance of ANANSE using experimentally validated trans-differentiation strategies. **Figure S9.** Comparison of the top 10 key TFs predicted by different methods in seven experimentally validated trans-differentiation strategies. **Figure S10.** Classification of the human gene expression and TF influence score.

Additional file 2: Table S1. Overview of accessions of public data that was used.

Additional file 3: Table S2. Summary of genomic locations of TF binding sites

Additional file 4: Table S3. Trans-differentiation expression dataset

Additional file 5: Table S4. Differential gene expression results for cell types used the trans-differentiation benchmark.

Additional file 6: Table S5. Trans-differentiation key TFs prediction results among different network sizes

Additional file 7: Table S6. Trans-differentiation key TFs prediction results among enhancer promoter and expression network

Additional file 8: Table S7. Application of ANANSE to human tissue data

Additional file 9: Supplementary Note S1. Detailed description of the GRN benchmarking procedure.

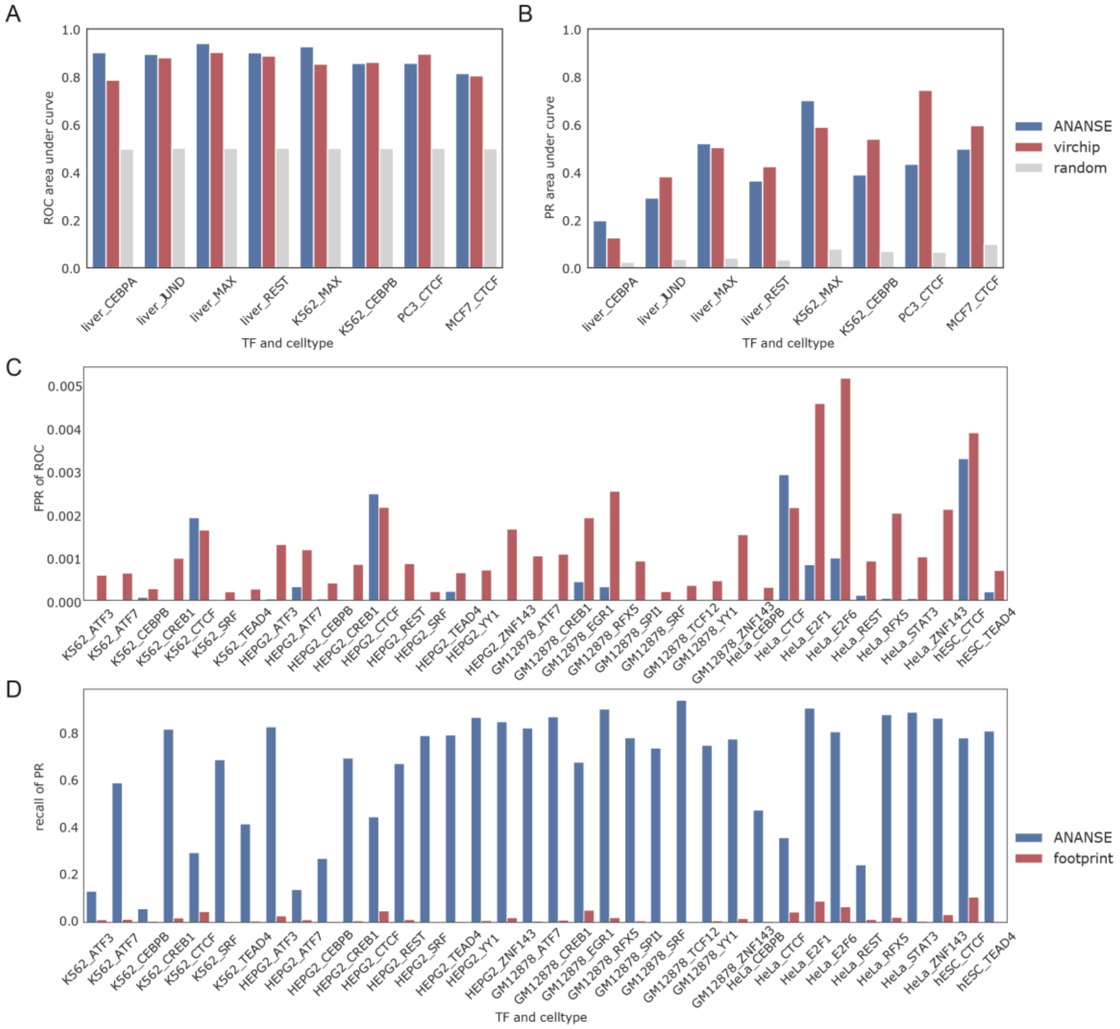

Supplementary Figure S1. Comparison of ANANSE TF binding prediction performance to other methods.

(A) The barplot shows ROC AUC of TF binding prediction of ANANSE compared to the Virtual ChIP-seq predictions in the ENCODE-DREAM validation cell types. ROC AUC of the ANANSE predicted TF binding is shown in blue; the Virtual ChIP-seq predicted result in red and the random model is indicated in gray. **(B)** The same evaluation as in A), with the PR AUC shown as a barplot. **(C)** Comparison of ANANSE binding predictions to DNAseI footprints obtained from (Vierstra et al., 2020). The barplot shows False Positive Rate (FPR) of TF binding prediction of ANANSE (blue) compared to the footprint TF binding prediction (ref) in ENCODE-DREAM validation cell types. The FPR is shown at the selected TPR of the footprints. **(D)** The same evaluation as in C), with the Recall shown as a barplot. The recall for ANANSE is calculated at the same precision of the footprint-based TF binding prediction.

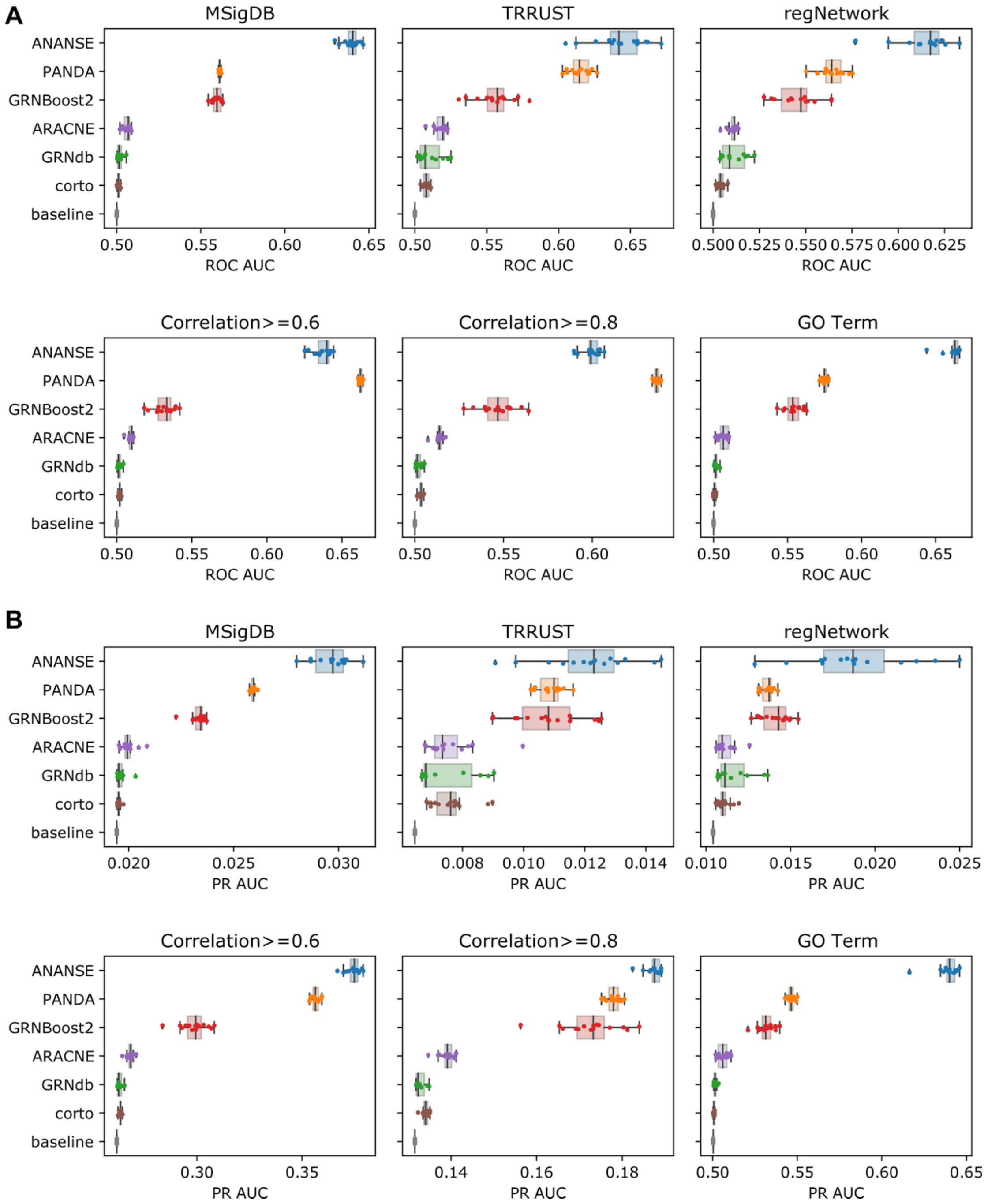

Supplementary Figure S2. Evaluation of tissue-specific enhancer GRNs predicted by ANANSE.

(A) Evaluation of the predicted networks, similar to Figure 4A and 4B, using different types of data: two TF-Gene regulatory networks based on interaction databases (MsigDB, TRRUST, regNetwork, Correlation>=0.6, Correlation>=0.8, and GO Term). The boxplots show the AUC of ROC for 15 different tissues. ROC AUC of the ANANSE predicted networks is shown in blue; the PANDA networks in orange; the GRNdb networks in green; the GRNBoost2 networks in red; the ARACNE networks in puple; the corto networks in brown; the random baseline networks in gray. (B) The same evaluation as in A), with the PR AUC shown as a boxplot.

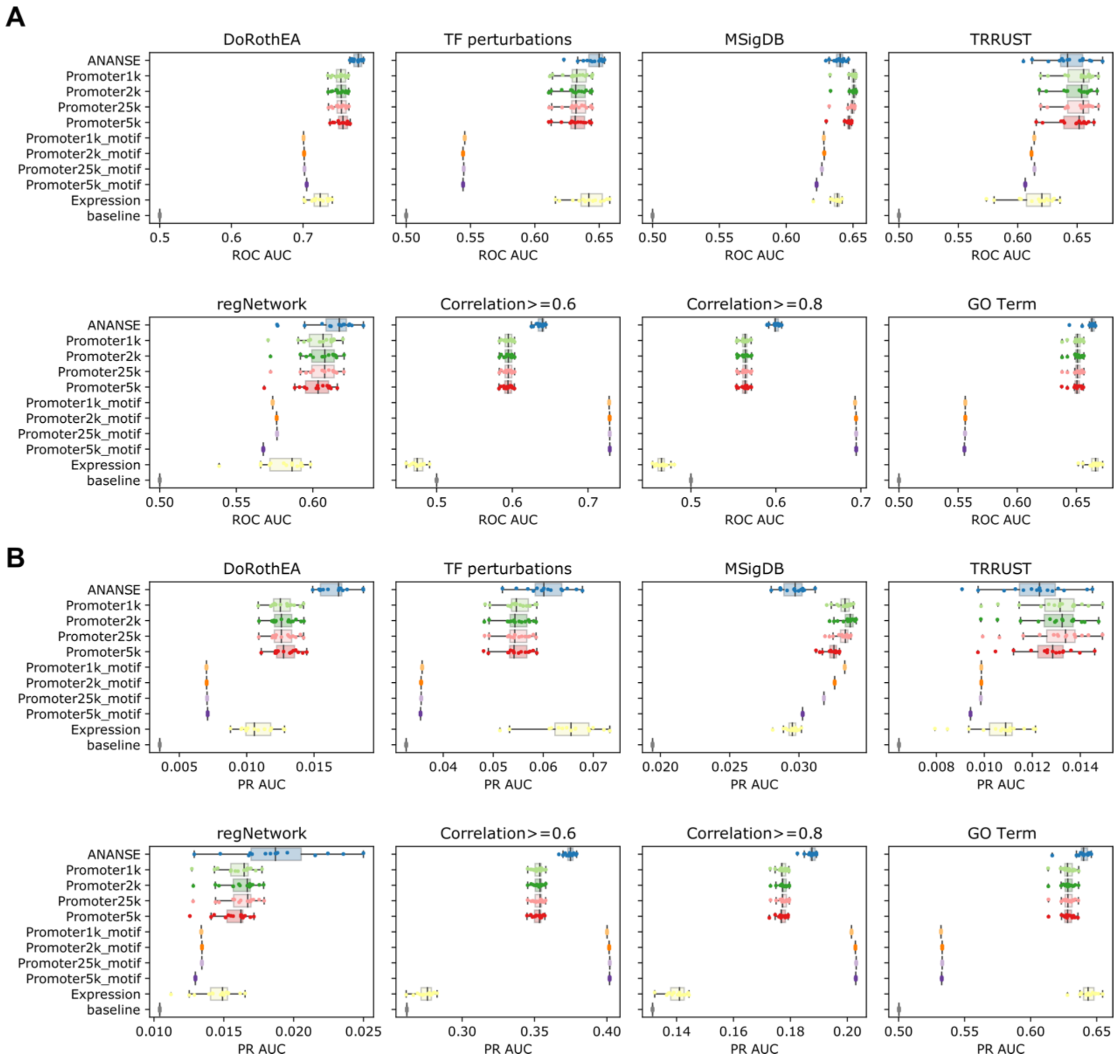

Supplementary Figure S3. Evaluation of predicting tissue-specific GRNs based on enhancer, promoter or expression data.

(A) The boxplots show the evaluation of four types of networks: ANANSE (the full model), PromoterXK (ANANSE based on promoters, instead of enhancers), PromoterXK_motif (GRN inference based on motif score alone) and Expression (GRN inference based on expression of TF and target gene alone). Four definition of promoters were tested, based on the distance around the gene TSS: 1kb, 2kb, 5kb and 25kb. The networks were evaluated based on the same references as Fig. 4 and Sup. Fig S2. Shown is the ROC AUC of the tissue-specific networks compared to the reference data. (B) The same evaluation as in A), with the PR AUC shown as a boxplot.

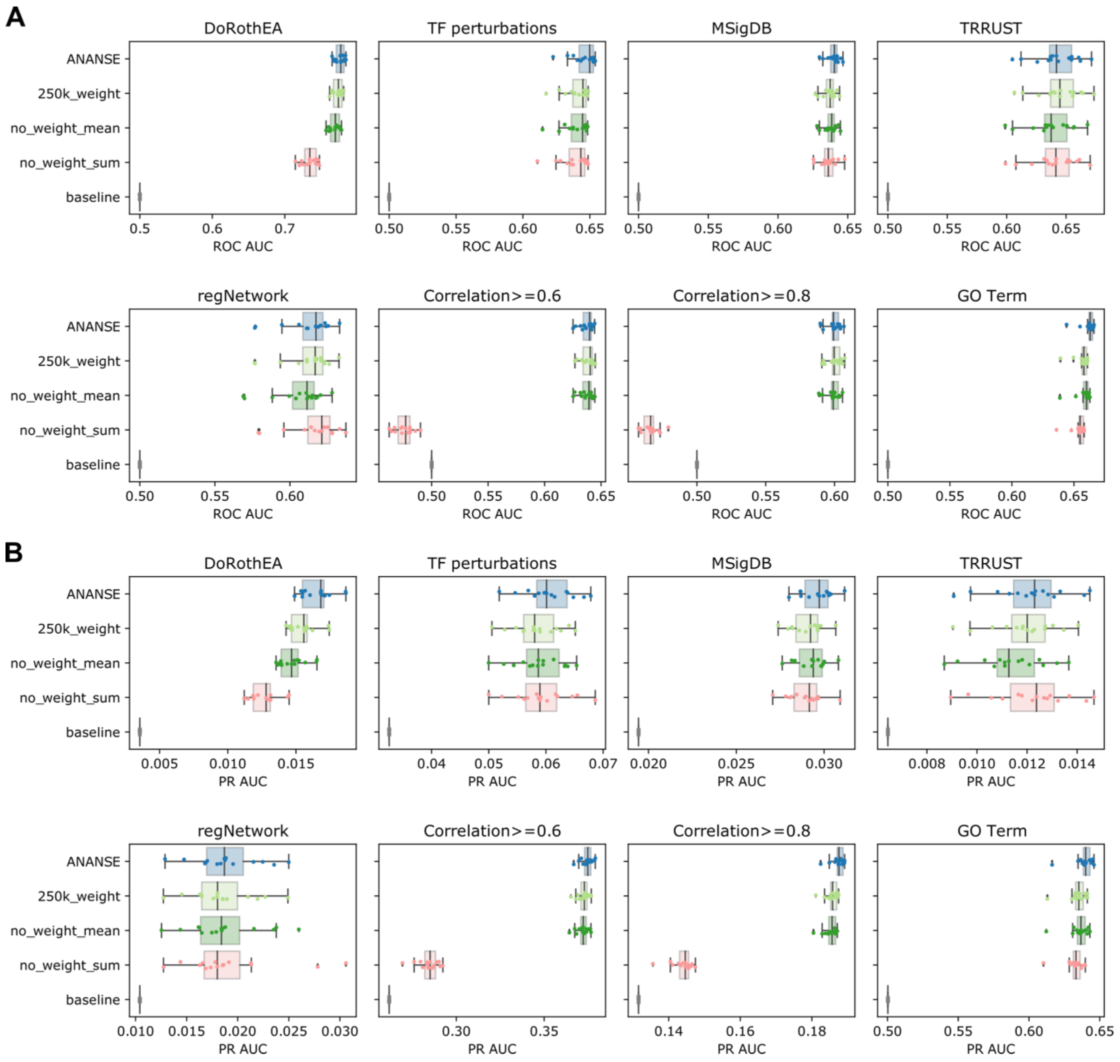

Supplementary Figure S4 Evaluation of tissue-specific enhancer GRNs with enhancer-gene summarization methods.

(A) Evaluation of the predicted GRNs, as described before. The plots show four different methods of calculating the TF-gene binding score: ANANSE, 250k_weight (the distance-weighted sum with enhancers within 250kb instead of 100kb of the gene TSS), no_weight_mean (the mean of all scores within 100kb, with equal weight) and no_weight_sum (the sum of all scores within 100kb, with equal weight). The boxplots show the AUC of ROC for 15 different tissues. ROC AUC of the ANANSE predicted networks is shown in blue; the 250kb weighted enhancer networks in light green; the 100kb mean enhancer networks in green; the 100kb sum enhancer networks in light red; the random baseline networks in gray. (B) The same evaluation as in A), with the PR AUC shown as a boxplot.

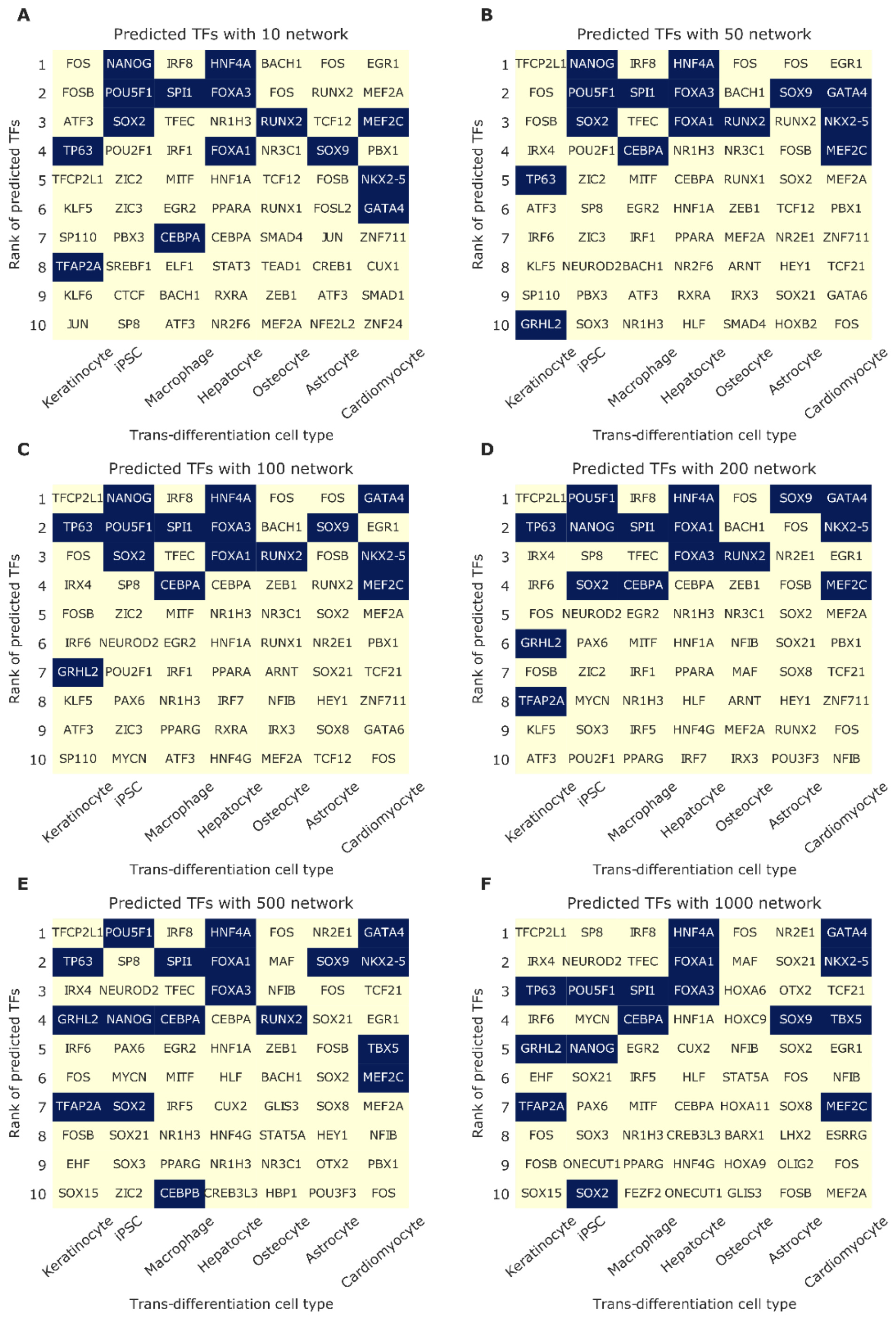

Supplementary Figure S5. Comparison of the top 10 key TFs predicted by different GRN sizes in seven experimentally validated trans-differentiation strategies.

The x-axis shows seven experimentally validated trans-differentiations, and the y-axis shows the top 10 predicted key TFs ranked by their influence score. Black boxes highlight the TFs that were used in trans-differentiation experiments. **(A)** 10k network. **(B)** 50k network. **(C)** 100k network. **(D)** 200k network. **(E)** 500k network. **(F)** 1000k network.

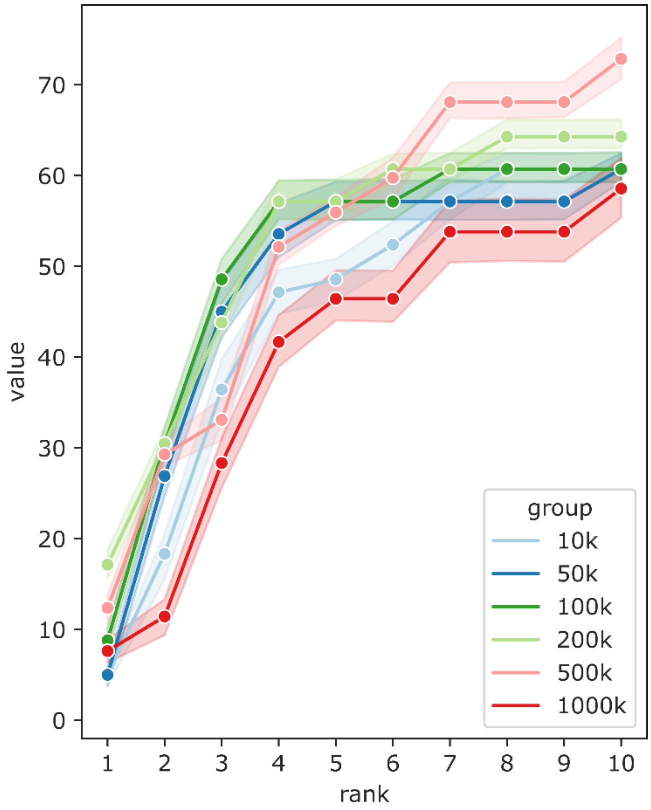

Supplementary Figure S6. Comparison of different GRN sizes used in the ANANSE prediction in seven experimentally validated trans-differentiation strategies.

(A) The line plots show the comparison of the predicted key TFs for six different sizes of GRNs Shown is the fraction of predicted TFs compared to all known TFs based on trans-differentiation protocols described in the literature (y-axis) as a function of the top number of TFs selected (x-axis). The shaded area represents the minimum and maximum percentage of corresponding recovered TFs when using six out of seven trans-differentiations.

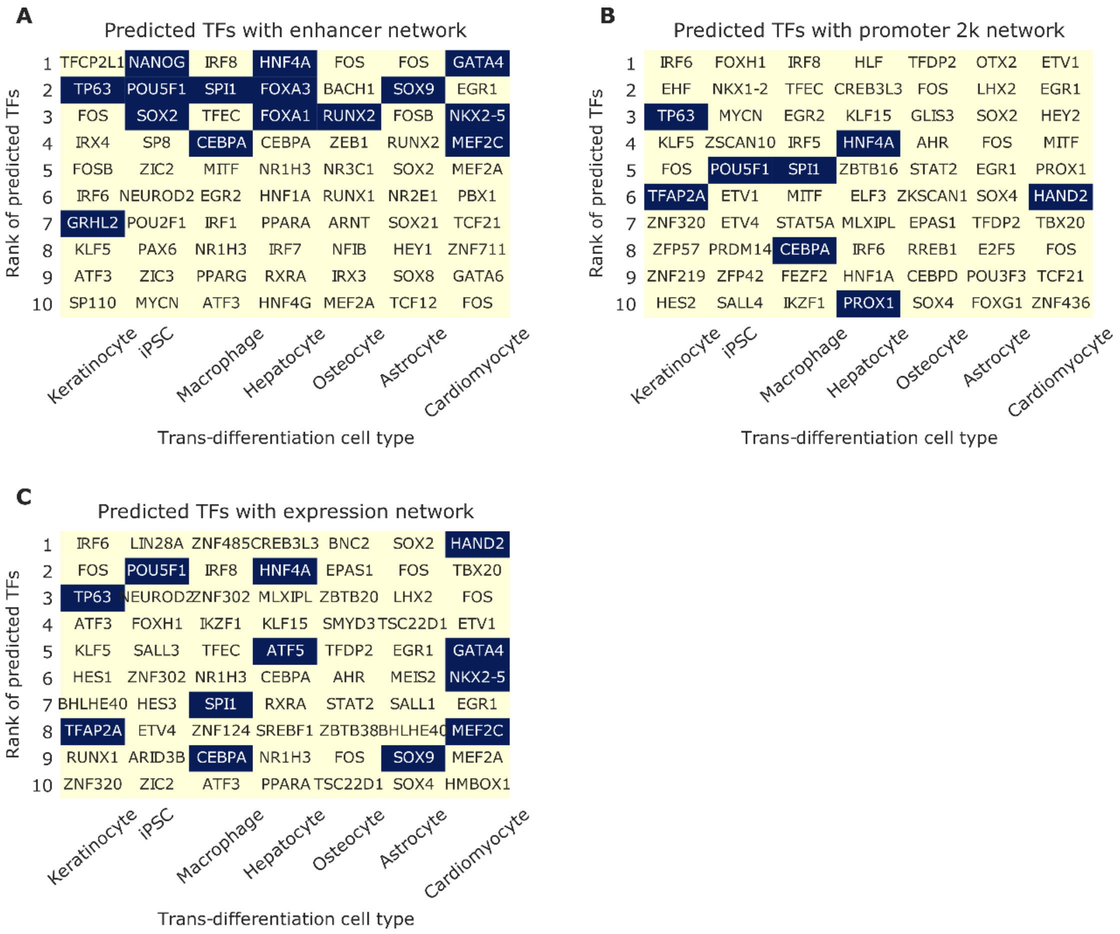

Supplementary Figure S7. Comparison of the top 10 key TFs predicted by different GRNs (enhancer, promoter and expression) in seven experimentally validated trans-differentiation strategies.

The x-axis shows seven experimentally validated trans-differentiations, and the y-axis shows the top 10 predicted key TFs ranked by their influence score. Black boxes highlight the TFs that were used in trans-differentiation experiments. **(A)** The results for ANANSE, based on a GRN that was inferred using peaks, regardless of their genomic location (includes both promoters and enhancers). **(B)** The results for ANANSE, based on a GRN that was inferred using the highest peak in the gene promoter, defined as < 2kb from the gene transcription start site. **(C)** The results for ANANSE, based on a GRN that was inferred using only the expression levels of the TFs and target genes.

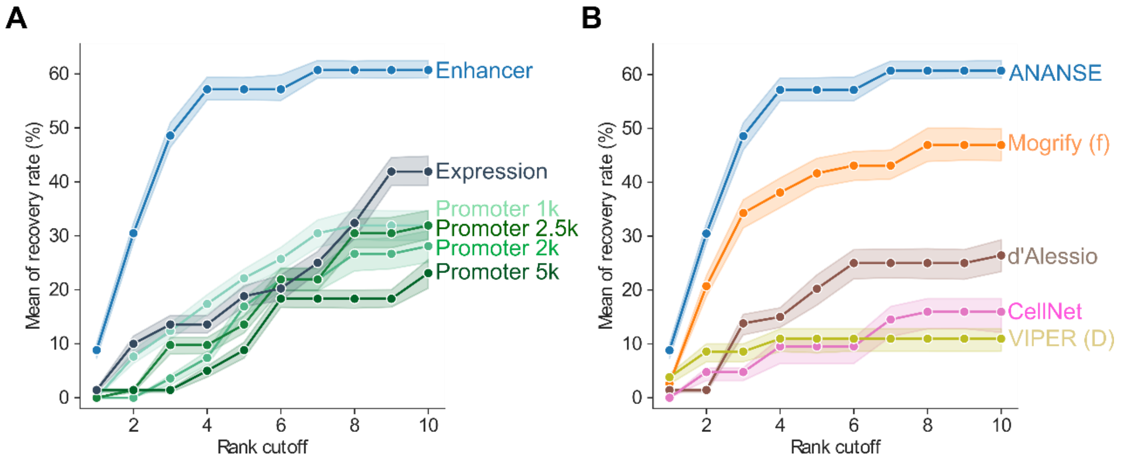

Supplementary Figure S8. ***Evaluation of the performance of ANANSE using experimentally validated trans-differentiation strategies.***

(A) The line plots show the comparison of the predicted top TFs for trans-differentiation from cell type-specific networks. Based on the difference between two networks, TFs were prioritized using the influence score calculation implemented in ANANSE. Shown is the fraction of predicted TFs compared to all known TFs based on trans-differentiation protocols described in the literature (y-axis) as a function of the top number of TFs selected (x-axis). The mean of recovery rate is the average of all TF sets when corresponding trans-differentiation has server different experimental validate TF sets. The shaded area represents the minimum and maximum percentage of corresponding recovered TFs when using six out of seven trans-differentiations. Three different types of networks were used: gene expression (deep green), promoter-based (promoter in 1kb, 2kb, 2.5kb, and 5kb) TF binding in combination with expression (blue), and enhancer-based TF binding in combination with expression (blue). (B) The line plots show the comparison of the predicted top TFs for trans-differentiation based on different computational methods. The y-axis indicates the percentage of experimentally validated cell TFs that are recovered as a function of the number of top predictions, similar as in A). Six different methods are shown: ANANSE (blue), Mogrify (full) (orange), d'Alessio (brown), CellNet (red), and VIPER with Dorothea network (yellow). The shaded area represents the minimum and maximum percentage of corresponding recovered TFs when using six out of seven trans-differentiations. CellNet only contains data from fibroblast to ESC, Hepatocyte, and Macrophage; and Mogrify and CellNet only contain the top 8 predicted factors.

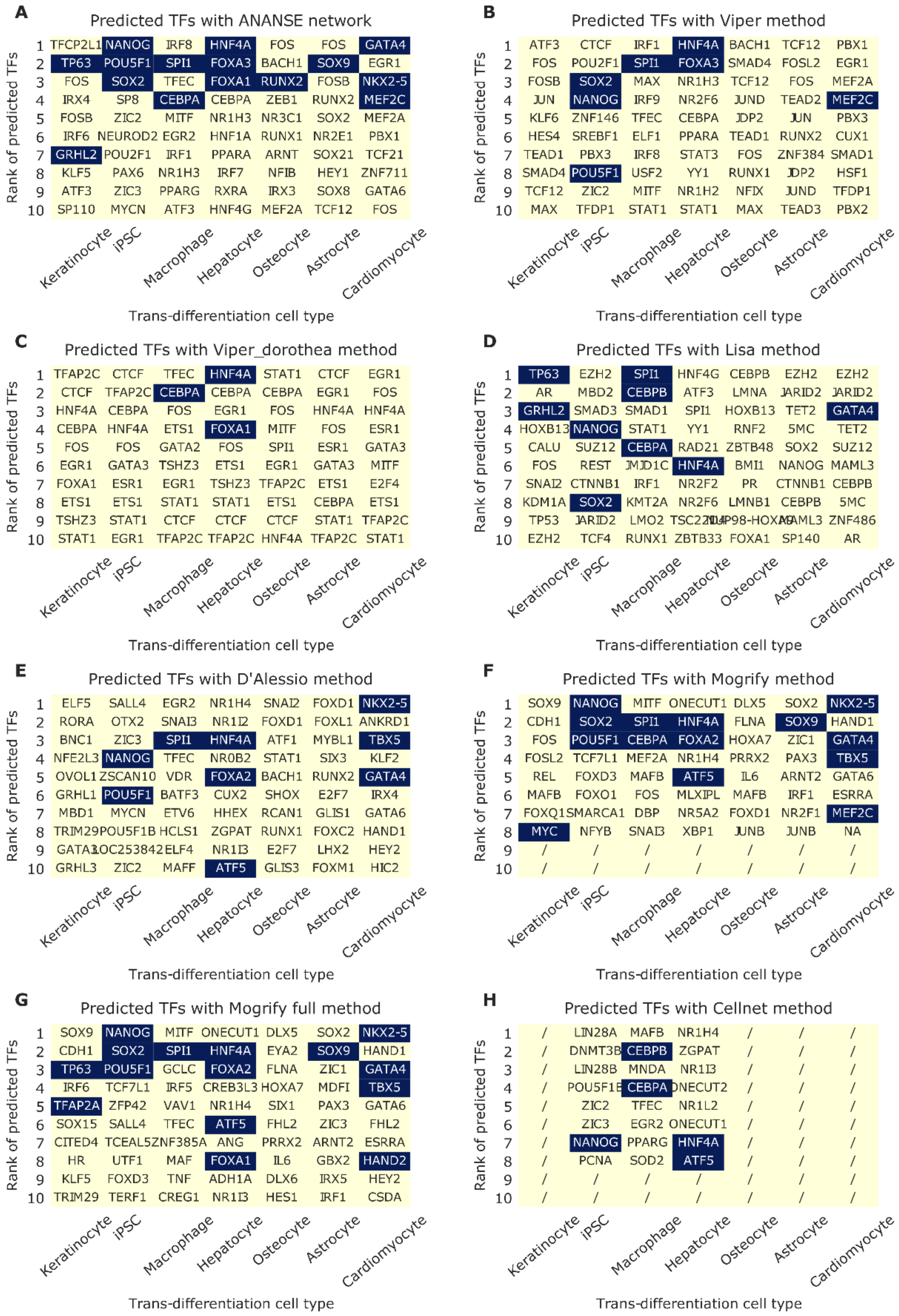

Supplementary Figure S9. Comparison of the top 10 key TFs predicted by different methods in seven experimentally validated trans-differentiation strategies.

The x-axis shows seven experimentally validated trans-differentiations, and the y-axis shows the top 10 predicted key TFs ranked by their influence score. Black boxes highlight the TFs that were used in trans-differentiation experiments. **(A)** ANANSE. **(B)** VIPER with ANANSE-predicted networks. **(C)** VIPER with DoRothEA networks **(D)** LISA **(E)** D'Alessio. **(F)** Mogrify. **(G)** Mogrify full list. **(H)** CellNet.

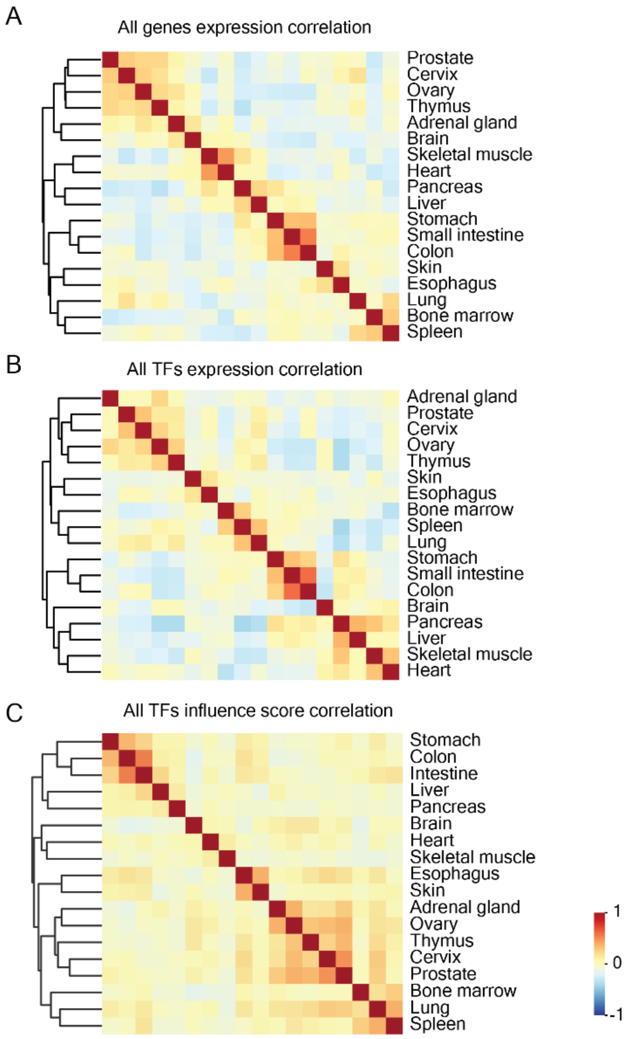

Supplementary Figure S10. Classification of the human gene expression and TF influence score.

(A), Heatmap showing the pairwise correlation between all 18 tissues based on gene expression. The colors in the heatmap indicate high (red) or low (blue) correlation across the tissue set. **(B)**, Heatmap showing the pairwise correlation between all 18 tissues based on the expression of all TFs. **(C)**, Heatmap showing the pairwise correlation between all 18 tissues based on TF influence scores.
